# Novel Approach to Personalized Physician Recommendations Using Semantic Features and Response Metrics: Model Evaluation Study

**DOI:** 10.2196/57670

**Published:** 2024-08-15

**Authors:** Yingbin Zheng, Yunping Cai, Yiwei Yan, Sai Chen, Kai Gong

**Affiliations:** 1 Biomedical Big Data Center, The First Affiliated Hospital of Xiamen University, School of Medicine, Xiamen University Xiamen City China; 2 Meteorological Disaster Prevention Technology Center, Xiamen Meteorological Bureau Xiamen City China

**Keywords:** web-based medical service, text analysis, Sentence Bidirectional Encoder Representations From Transformers, SBERT, smart triage systems, patient-physician hybrid recommendation, PPHR, PPHR model

## Abstract

**Background:**

The rapid growth of web-based medical services has highlighted the significance of smart triage systems in helping patients find the most appropriate physicians. However, traditional triage methods often rely on department recommendations and are insufficient to accurately match patients’ textual questions with physicians’ specialties. Therefore, there is an urgent need to develop algorithms for recommending physicians.

**Objective:**

This study aims to develop and validate a patient-physician hybrid recommendation (PPHR) model with response metrics for better triage performance.

**Methods:**

A total of 646,383 web-based medical consultation records from the Internet Hospital of the First Affiliated Hospital of Xiamen University were collected. Semantic features representing patients and physicians were developed to identify the set of most similar questions and semantically expand the pool of recommended physician candidates, respectively. The physicians’ response rate feature was designed to improve candidate rankings. These 3 characteristics combine to create the PPHR model. Overall, 5 physicians participated in the evaluation of the efficiency of the PPHR model through multiple metrics and questionnaires as well as the performance of Sentence Bidirectional Encoder Representations from Transformers and Doc2Vec in text embedding.

**Results:**

The PPHR model reaches the best recommendation performance when the number of recommended physicians is 14. At this point, the model has an *F*_1_-score of 76.25%, a proportion of high-quality services of 41.05%, and a rating of 3.90. After removing physicians’ characteristics and response rates from the PPHR model, the *F*_1_-score decreased by 12.05%, the proportion of high-quality services fell by 10.87%, the average hit ratio dropped by 1.06%, and the rating declined by 11.43%. According to whether those 5 physicians were recommended by the PPHR model, Sentence Bidirectional Encoder Representations from Transformers achieved an average hit ratio of 88.6%, while Doc2Vec achieved an average hit ratio of 53.4%.

**Conclusions:**

The PPHR model uses semantic features and response metrics to enable patients to accurately find the physician who best suits their needs.

## Introduction

### Background

Web-based medical consultation is increasingly popular as an alternative to traditional health care services because it is convenient, accessible, and affordable [1]. This type of patient-physician interaction takes place electronically, connecting both parties through text, images, and videos. Its advantages include eliminating time and space constraints and accurately documenting the medication process [2], making it more attractive to many patients than in-person medical visits. As of the end of 2022, the number of users in China’s internet medical and health market reached 363 million [3]. The rapid growth of web-based medical services and the vast amount of information available have created considerable difficulties for patients in finding the physicians best suited to their needs, leading to potentially mismatched consultations [4].

At present, most existing triage procedures rely on manual recommendation from schedulers to select departments for patients. As the number of consultations increases, manual provision of advice does not guarantee the professionalism and quality of medical services [5]. In addition, schedulers are unable to provide 24-hour service, resulting in gaps in health care access and the continuity of services. At this point, a common approach might be to develop an intelligent department recommendation model. Advancements in technology, particularly in the field of machine learning, present opportunities to improve the accuracy and efficiency of patient department assignment in health care systems. For example, Mullenbach et al [6] integrated the attention mechanism and used long short-term memory to predict the patient’s disease type for further triage. Li and Yu [7] used multifilter residual convolutional neural networks to investigate the issue of department recommendation. Wang et al [8] used the Bidirectional Encoder Representations from Transformers (BERT) model to study disease diagnosis and department recommendations. These approaches can potentially automate the process of assigning patients to appropriate departments, reducing the burden on schedulers and improving patient outcomes through more accurate and timely care.

However, due to the ongoing subdivision of departments, these department recommendation models still cannot accurately match medical needs with physicians’ specialties. For example, obstetricians and gynecologists further specialize in subfields such as gynecology, obstetrics, reproductive endocrinology, infertility, prenatal diagnosis, and genetic counseling. This refined division not only improves the effectiveness of diagnosis and treatment but also ensures that patients receive the most cutting-edge and professional care plans. In addition, even if the diseases treated are similar or the same, different medical institutions may have different department names. These problems have placed higher demands on hospital management, requiring more precise resource allocation to adapt to increasingly specialized services. Therefore, there is an urgent need to design personalized physician recommendation models.

Personalized recommendation methods can help users manage massive amounts of information and knowledge [9] and are crucial for providing personalized medical services that meet the patient’s needs [10]. For instance, Ju and Zhang [11] integrated geographical location and patients’ questions to generate personalized recommendations. Liu et al [12] proposed a physician recommendation model that considers the characteristics of patients and physicians. Lu et al [5] proposed a self-adaptive physician recommendation system that considers physician activity and patient feedback. These methods can be advantageous for both patients and web-based health care providers, as they minimize the time and effort required to find a suitable match, thus ensuring efficient delivery of health care services [13].

However, there are still some shortcomings in previous studies. Most existing studies use satisfaction as a measure of physician performance. However, the authenticity of satisfaction ratings across different platforms is not always reliable, as many users tend to habitually provide positive feedback. In terms of the evaluating indicators for recommended physicians, most studies used accuracy as a single indicator and did not consider the service quality of recommended physicians. These limitations may result in consultation mismatches, longer patient waiting times, and potentially reduced patient satisfaction. To the best of our knowledge, previous studies have not developed a triage system for recommending physicians that uses the transformer-based models, which are the cutting-edge models for natural language processing. BERT [14] is a popular transformer-based model that has been pretrained on common texts, such as Wikipedia and the Brown Corpus. BERT is a state-of-the-art model that uses an attention-based mechanism [15,16] to accurately understand the context of words, enabling unsupervised learning by linking text input and output through a decoder-encoder framework [17,18]. However, the BERT model is not suitable for semantic similarity searches or clustering, which has led to the creation of a different sentence-embedding model called the Sentence BERT (SBERT) model [19]. This modified version of the BERT model was designed to be semantically meaningful and suitable for sentence similarity tasks. It works by integrating a Siamese network and a pretrained BERT model, along with a pooling layer that generates a fixed-sized representation. The SBERT model can accurately identify whether there is a significant match between 2 sentences, making it a useful tool for data mining, information retrieval, and text matching [20].

### This Study

The objective of this study was to develop a more precise algorithm that can better recommend professional and highly engaged physicians and thus improve the effective use of medical resources and the medical experience of patients by reducing the mismatches between medical needs and services. The practical benefits expected from our findings include the enhanced ability of web-based health care platforms to provide timely, relevant, and professional medical consultations that are closely tailored to each patient’s unique needs. By implementing our advanced recommendation algorithm, we expect to not only identify the most appropriate specialists based on patient input but also incorporate a comprehensive evaluation of physician performance metrics. This will ensure that patients are recommended physicians who are not only experts in their field but also highly engaged and responsive, resulting in higher-quality care.

We seek to answer these three questions: (1) how can we effectively construct features for patients and physicians to facilitate efficient physician recommendations? (2) how can we incorporate the physicians’ performance metrics into recommendation strategies to increase the chance of recommending highly active physicians? and (3) how can the effectiveness of the recommendation strategy be verified considering both accuracy and service quality?

## Methods

### Data Collection

This research collected a total of 646,383 web-based medical consultation records from the Internet Hospital of the First Affiliated Hospital of Xiamen University between 2016 and 2023. Each record contains the textual question, deidentified codes for the physician and patient, the physician’s department, and the response status and time. Response status refers to whether a consultation request has received a reply from the corresponding physicians. Response time is the duration between submitting a request and getting a response. A total of 5 examples of the questions generated during web-based medical consultations are displayed in Table 1.

These records were divided into 2 test data sets and 1 training data set. For the first test data set, the physician with the highest number of consultations was selected from each of the following departments with the most inquiries: gastroenterology, obstetrics, respiratory medicine, pediatrics, and dermatology. Their codes were 98, 141, 202, 512, and 601, respectively. A total of 400 consultation records were randomly selected from each of the aforementioned physicians. These physicians then reviewed these textual questions to determine whether they were within their expertise. Any questions that a physician is proficient in was tagged, and eventually we randomly selected 200 records for each physician from these tagged questions to compile a test data set consisting of 1000 records. For the second test data set, a sample of 10,000 consultations was randomly chosen from the total data set, excluding the consultation samples from the first test data set. The training data set consisted of the consultations remaining after the removal of the first and second test data sets. The random seed for this study was set to 2023.

**Table 1:** Examples of patients’ consultation questions.

Sample number	Patient code	Questions	Physician code	Department	Response status^a^	Response time
1	200321	You initially diagnosed me with left varicocele and ordered a color Doppler ultrasound examination. The results showed a moderate left varicocele with reflux. I would like to inquire whether, aside from surgery, this condition can be treated through medication, injections, or other noninvasive methods?	208	Urology	True	8 h 12 min
2	306878	My child has been experiencing discomfort in the throat and recurring fevers for four days before visiting a physician, who considered it was pneumonia and started administering azithromycin. Today is the fifth day of treatment. After the first day of intravenous azithromycin, the fever subsided, but there is still occasional coughing with phlegm. I am worried about the potential for significant side effects. Should the child continue taking azithromycin?	372	Pediatrics	False	null
3	447138	I am currently on my period and have scheduled an ultrasound and mammography for this afternoon. Could you please tell me if there is a recommended waiting period before trying to conceive after a mammography?	133	Breast surgery	True	20 min
4	591872	What does the glucose tolerance test report indicate? Could you please explain it to me?	423	Obstetrics	True	9 min
5	603826	My chin is red without feeling painful or itchy, and it has been like this for over a month. I’ve tried Clotrimazole but no obvious effects were achieved. Could you please tell me what condition this might be and what medication I should use?	418	Dermatology	True	2 h 20 min

^a^True: the physician has responded to the consultation request; false: the physician has not responded to the consultation request.

### Data Preprocessing

Data related to patients’ consultation questions were collected and presented in the form of natural language. Preprocessing of these unstructured data is crucial in machine learning framework [21] to remove unnecessary, duplicated, irrelevant, and noisy data [22]. This study involved several steps to process these consultation questions, including normalization, tokenization, part-of-speech tagging, and stop-word removal, thereby forming a reliable corpus.

The study calculated response rates and times for all physicians as shown in equations 1 and 2, where *N*_R_ denotes the number of consultation requests that physician *P*_i_ has responded to, with “responded” indicating that the response status is confirmed as true. Meanwhile, *N* indicates the total number of consultation requests that physician *P*_i_ has received. Furthermore, *S*_T_ refers to the total response time for all the consultation requests that physician *P*_i_ has responded to:



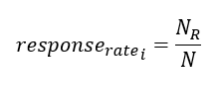



**(1)**



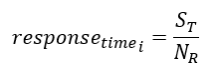



**(2)**

Upper and lower bounds on response times were established to minimize the impact of extremely high and low values on the experiment. Response times >95% were capped at 8 hours and 6 minutes, while those below the fifth percentile were raised to 9 minutes.

### Feature Extraction

Feature extraction is the process of converting raw input data into a meaningful set of features [23] that can be understood by machine learning classifiers. In the feature extraction stage, 2 unique features for both patients and physicians were introduced.

#### Patients’ Feature Modeling

**Figure 1:**
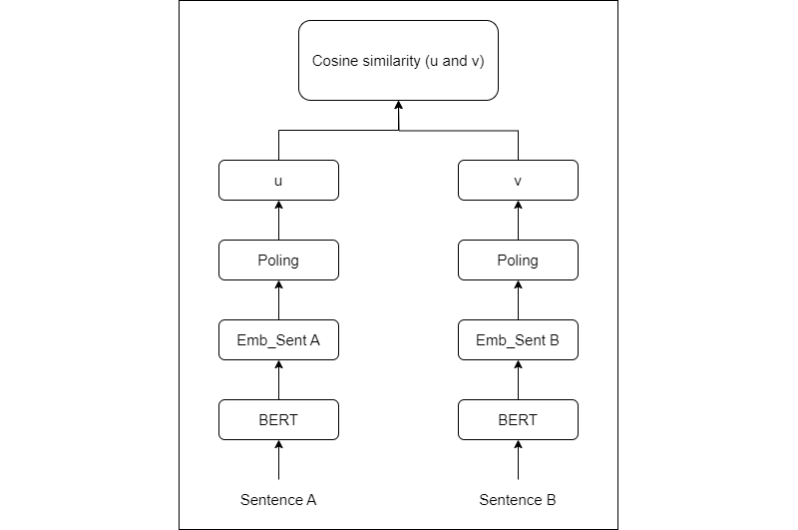
Sentence Bidirectional Encoder Representations from Transformers (BERT) architecture.

This study used the pretrained SBERT model known as “distiluse-base-multilingual-cased” to convert all consultation questions into semantic representations and then calculate sentence embeddings for further analysis. As shown in Figure 1 [19], the SBERT model processed sentences A and B through BERT pooling to generate their respective embeddings, *u* and *v*. The similarity between these embeddings is then calculated using the cosine similarity method, which effectively measures how similar sentences are. The cosine similarity is expressed by equation 3, where *u* and *v* represent 2 vectors:



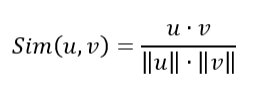



**(3)**

#### Physicians’ Feature Modeling

Term frequency–inverse document frequency (TF-IDF) model [24,25] is a commonly used method in text mining and information retrieval because it can capture the importance of words and has the potential to extract features from multiple texts. The formula of this algorithm is shown in equation 4:



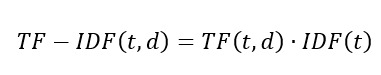



**(4)**

where *TF* (*t,d*) represents the frequency of a specific keyword *t* in document *d*, while *IDF* (*t*) signifies the inverse document frequency. According to this formula, the higher the *TF-IDF* (*t,d*) value, the more significant the feature is in the document.

This study used the TF-IDF model to extract crucial information from a collection of patients’ consultation questions aggregated by physician codes, selecting the top 20 with the highest TF-IDF weights. This extracted information was then fed into an SBERT model to compute cosine similarity among physicians.

### Recommendation

#### Overview

**Figure 2:**
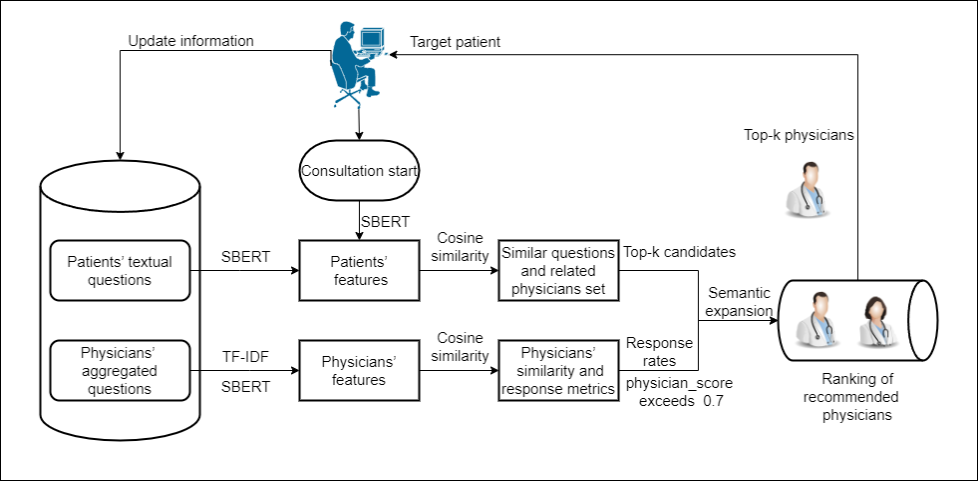
The architecture of the patient-physician hybrid recommendation model. SBERT: Sentence Bidirectional Encoder Representations from Transformers; TF-IDF: term frequency–inverse document frequency.

A patient-physician hybrid recommendation (PPHR) model with response metrics was developed by combining features of both patients and physicians. This model also considers the physician’s response rate in the recommendation strategy. The PPHR model, which is a type of top-*k* recommendation system, is designed to provide patients with a list of the top-*k* physicians who are most likely to meet their medical needs, as illustrated in Figure 2.

#### Step 1: Generate a Candidate Set Using the Patient Feature–Based Model

The patient feature–based (PFB) model was developed to identify sets of the most similar questions. When a new consultation starts, the consultation questions will be processed using SBERT to generate the corresponding embeddings. The patients’ features were used to construct a similarity matrix among questions using cosine similarity. Then, consultation questions that are similar to the new consultation will be identified by comparing the patients’ features. The physicians who are associated with these similar questions are considered potential candidates. The similarity score, known as the init_score, serves as the baseline for making recommendations. The top-*k* physicians are selected as candidates from the set of similar questions, where *k* is an adjustable hyperparameter in this model.

#### Step 2: Expand the Candidate Set Based on the Patient-Physician Hybrid Model

As patients’ textual questions are unprofessional, setting a similarity threshold based solely on the patient characteristics may limit the recommendation results. The patient-physician hybrid (PPH) model ensures that all potential physician recommendations are considered. This model is formed by combining the physicians’ features with the PFB model to semantically expand the scope of candidates. It does this by creating an index called the expand_score, which is derived from the physicians’ features. This index reflects the degree of similarity among physicians and helps determine which physicians have the necessary expertise and qualifications to provide the right care for a given patient. This approach can adjust biases in the system that may arise from recommending physicians based solely on similarities to patients’ questions. The PPH model is shown in equation 5:

physician_score_i_ = init_score_i_ × expand_score_i_ **(5)**

If the physician_score exceeds 0.7, it will be used to semantically expand the range of candidates. When the physician is not derived from the PPH model, the expand_score is assumed to be 1.

#### Step 3: Optimize the Ranking of the Candidate Set by Incorporating the Response Rate

The response rate can serve as an indicator to measure the efficacy of physicians’ performance. An increase in the response rate suggests that physicians are more willing to treat patients. This can be viewed as a positive feedback loop, as higher response rates lead to more motivated physicians. Therefore, it is crucial to consider the physicians’ activity level along with the similarity index, as this can skew the recommendation results toward more active physicians, increasing the chance that inquiries will be answered and thus improving patient satisfaction. The final PPHR model is displayed in equation 6. The top-*k* physicians are selected for recommendation based on the scores, where *n* represents the number of times physician *D*_i_ is recommended:



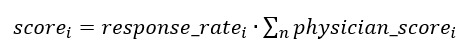



**(6)**

### Evaluation

The proposed PPHR model’s effectiveness was evaluated using the following metrics: hit ratio, precision, recall, *F*_1_-score, and high-quality service proportion. In the first test data set, a recommendation was considered correct when the selected physician was among the top-*k* recommended physicians. The hit ratio refers to the proportion of correct recommendations to the total number of recommendations. In the second test data set, a recommendation was regarded as accurate if the recommended physician’s maximum physician_score is >0.7. Precision refers to the proportion of correctly recommended physicians to the total number of recommended physicians. By contrast, recall is the ratio of correctly recommended physicians to the number of physicians who should have been retrieved in the sample. The *F*_1_-score is a valuable metric for assessing the recommendation algorithm’s effectiveness, as it merges precision and recall to yield the best results. A higher *F*_1_-score signifies a more efficient algorithm. Precision, recall, and *F*_1_-score were calculated using the formulas described in equations 7 to 9, where *TP* is a true positive, *FP* is a false positive, and *FN* is a false negative:



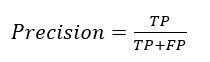



**(7)**



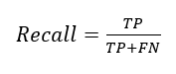



**(8)**



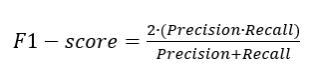



**(9)**

A quick response time is a critical element for high user satisfaction, allowing the system to promptly provide services that meet patient expectations. If a physician responds quickly, the patient will perceive the quality of the physician’s service to be better than that of a physician who takes a longer time to respond. Therefore, the proportion of physicians who respond quickly among all recommended physicians, known as the high-quality service proportion, is a significant measure of evaluation. The calculation for high-quality service proportion is determined by the formula shown in equation 10:



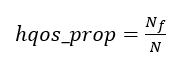



**(10)**

The term *N*_f_ represents the number of physicians whose response time is faster than the average response time, while *N* denotes the number of recommended physicians.

### Baseline Experiments

For the PFB model, the purpose of baseline experiments was to determine whether excluding physicians’ features from the PPHR model would degrade performance. A total of 3 steps were taken to assess its performance compared to the PPHR model. First, the hit ratio and ranking of the selected physician in the recommendation set were calculated in the first test data set. Second, the precision, recall, and *F*_1_-score of the recommendation results were computed in the second test data set, and the consultation questions that were recommended to the selected physician (codes 98, 141, 202, 512, and 601) were collected. Finally, a questionnaire for assessing the rationality of the recommendations was administered by randomly selecting 200 consultation questions (100 for each model) for each physician from those consultation questions. The questionnaire included an evaluation of the relevance of each selected physician with the consultation questions. The survey question was as follows: “Based on your area of expertise, how would you rate the match between you and consultation question?” The questionnaire used a Likert 5-point scale [26] for measurement, with scores ranging from 1 (very inappropriate) to 5 (very appropriate). The Mann-Whitney *U* test [27] was used to determine whether there was a statistical difference in the physicians’ perceptions of how well the consultation questions from these 2 models matched their area of expertise.

For the PPH model, the proportion of high-quality services in the recommendation results was calculated to assess whether eliminating the response rate in the PPHR model will reduce service quality.

Doc2Vec [28] was used to create text embeddings for all patients’ consultation questions to reconstruct the PPHR model. The performances of Doc2Vec against SBERT were evaluated in the first test data set to determine the effectiveness of transfer learning without contextual modeling. The model’s performance is measured by the hit ratio and the ranking of the selected physician within the set of recommendations.

### Ethical Considerations

This study complied with all relevant ethical regulations. All the available data sets have been deidentified and anonymized. The First Affiliated Hospital of Xiamen University Ethics Committee approved this study (approval number SL-2021KY044-01), and no informed consent was necessary.

## Results

### Data Set Summary

Among the 646,383 consultation records, there were 193,675 patients and 858 physicians across 44 departments. According to Table 2, which provides a summary at the record level, 32.95% (n=212,983) of the records were created by male patients, while female patients accounted for 67.05% (n=433,400) of the records. The predominant age group among patients was 20 to 39 years, representing 54.6% (n=352,907) of the total number of consultations. Patients most frequently consult senior physicians, who account for 62.65% (n=404,958) of all consultations. Most consultations were initiated between 12 and 17 hours, accounting for 37.04% (n=239,401) of the total, while the bulk of responses were received between 18 and 23 hours, accounting for 40.94% (n=208,417). The average response time of the physicians was 3 hours and 40 minutes, with an average response rate of 65.2% (n=421,441).

**Table 2:** Summary of the characteristics of the collected data records (N=646,383).

Characteristic		Value, n (%)
**Gender**		
	Male	212,983 (32.95)
	Female	433,400 (67.05)
	Intersex	0 (0)
**Age group (y)**		
	<20	118,484 (18.33)
	20-39	352,907 (54.6)
	40-59	125,957 (19.49)
	>60	49,035 (7.58)
**P** **hy** **sician** **s’ professional title**		
	Junior	10,766 (1.67)
	Intermediate	45,892 (7.1)
	Subsenior	184,767 (28.58)
	Senior	404,958 (62.65)
**Consulted created moment (h)**		
	0-5	15,686 (2.43)
	6-11	195,297 (30.21)
	12-17	239,401 (37.04)
	18-23	195,999 (30.32)
**Consultation responded moment (h)**		
	0-5	13,394 (2.61)
	6-11	115,684 (22.73)
	12-17	171,634 (33.72)
	18-23	208,417 (40.94)
**Response status**		
	True	421,441 (65.2)
	False	224,942 (34.8)

### Case Analysis

This study recommends the following question, as shown in sample 4 in Table 1: “What does the glucose tolerance test report indicate? Could you please explain it to me?” This was done to verify the feasibility of the PPHR model. Questions similar to the target patient’s question and related candidate physicians are displayed in Table 3.

The physicians who were similar to the candidate physicians were identified and included in the recommendation strategy along with response indicators. The codes and scores of the recommended physicians of the PPHR model are displayed in Table 4.

To evaluate the precision of the recommended results, we compared the diagnoses for the consultation question within the recommended results. For example, physician 141’s diagnosis includes “gestational diabetes” and “glucose tolerance,” while physician 164’s diagnosis includes “glucose tolerance” and “diabetes.” This information matches the consultation question of sample 4, suggesting that the recommended results are likely accurate.

**Table 3:** Top-10 most similar questions to the target patient’s question and related physicians.

Question code	Cosine similarity	Physician code
394946	0.9766	178
559249	0.9765	423
317643	0.9618	141
409326	0.9419	164
238700	0.9214	456
2416	0.9004	707
173519	0.8990	304
551580	0.8976	330
466072	0.8906	632
93556	0.8861	391

**Table 4:** Top-10 recommended physicians.

Physician code	score	physician_score
141	7.1877	0.9618
164	5.9507	0.9419
423	4.1309	0.9765
456	4.0309	0.9214
166	3.9408	0.8978
178	3.2916	0.9766
169	3.2082	0.7851
181	2.7208	0.7310
335	2.7096	0.7672
189	2.4849	0.7358

### Evaluating the Effectiveness and Service Quality

**Figure 3:**
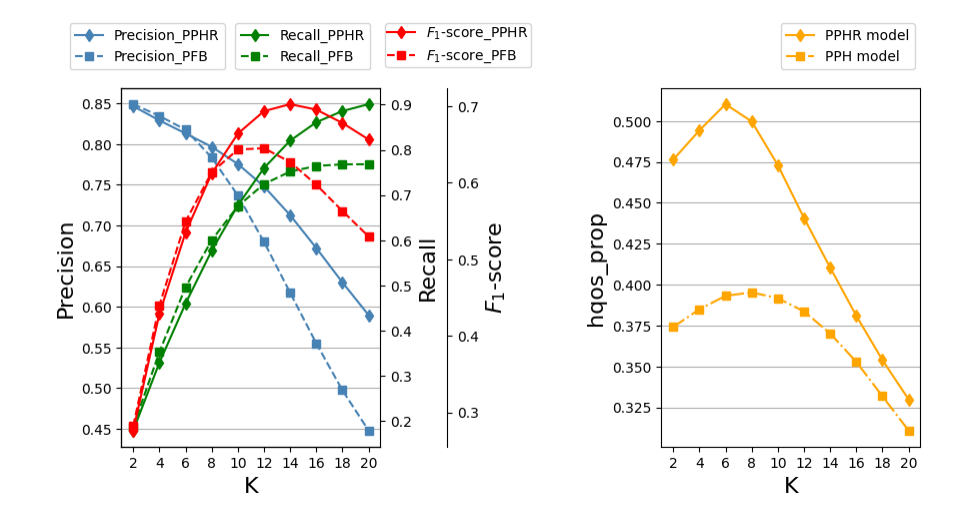
Comparison of our proposed patient-physician hybrid recommendation (PPHR) model with the patient feature–based (PFB) model and patient-physician hybrid (PPH) model in terms of various indexes, including precision (blue), recall (green), F1-score (red), and hqos_prop (orange).

Figure 3 shows a comparison of the PPHR model with the PFB and PPH models in terms of various indexes. The x-axis represents the number of recommended physicians (K), with values ranging from 2 to 20 and increasing in increments of 2. The PPHR and PFB models exhibit comparable performance when K is <10. However, as K increases to ≥10, the PPHR model demonstrates a marked improvement over the PFB model. The PPHR model achieves its highest *F*_1_-score when K is 14, indicating optimal performance at this level. At this stage, the model has a precision of 71.26%, a recall of 82.02%, and an *F*_1_-score of 76.25%. Compared to the PFB model, the precision has increased by 15.43%, the recall has increased by 9.10%, and the *F*_1_-score has increased by 12.05%. The results presented in Table 4 indicate that although there are minor fluctuations in the rankings of the selected physicians of the PPHR model, its hit ratio has increased by 2.19% compared to the PFB model. These indicate that incorporating physicians’ features into the recommendation strategy can improve the effectiveness of the recommendation system.

Regardless of the value of K, the PPHR model provides better hqos_prop than the PPH model. When K is set to 14, the high-quality service ratio of the PPHR model is 41.05%. This is an improvement of 10.87% over the PPH model. This suggests that incorporating the response rate into the recommendation strategy can enhance service quality.

### Evaluating the Performance of Text Embedding

The results displayed in Table 5 compare the SBERT model with the Doc2Vec model in terms of text embedding, with K set to 14. The ranking indicates the position of the selected physician within the recommendation set, while the hit ratio represents the percentage of successful recommendations. The SBERT model surpasses the Doc2Vec model by 65.92% in hit ratio, and the rankings of the selected physicians improved by 1.57. These findings suggest that using the SBERT model to create text embeddings could improve the performance of the recommendation system.

**Table 5:** Performance comparisons were conducted using different models for the selected physicians.

Physician code	Hit ratio			Ranking		
	Doc2Vec-PPHR^a^	SBERT^b^-PPHR	SBERT-PFB^c^	Doc2Vec-PPHR	SBERT-PPHR	SBERT-PFB
98	0.485	0.850	0.820	4.55	3.19	3.04
141	0.555	0.915	0.890	3.99	2.36	2.47
202	0.430	0.800	0.780	4.93	3.47	3.33
512	0.505	0.940	0.925	5.95	3.37	2.73
601	0.695	0.925	0.920	2.78	1.97	1.90
Combined, mean (SD)	0.534 (0.101)	0.886 (0.059)	0.867 (0.064)	4.44 (1.172)	2.87 (0.668)	2.69 (0.549)

^a^PPHR: patient-physician hybrid recommendation.

^b^SBERT: Sentence Bidirectional Encoder Representations from Transformers.

^c^PFB: patient feature–based.

### Rationality Evaluation

As can be seen in Table 6, for each physician, the PPHR model consistently received higher ratings than the PFB model. The average rating of the PPHR model is 3.90, which is 11.43% higher than that of the PFB model. The *P* values indicate that the differences in ratings between these 2 models are statistically significant, indicating that the PPHR model is capable of recommending better-performing physicians compared to the PFB model.

**Table 6:** Rationality evaluations of patient-physician hybrid recommendation (PPHR) model and patient feature–based (PFB) model for selected physicians.

Physician code	Rating		*P* value
	PPHR	PFB	
98	3.89	3.48	.01
141	4.00	3.57	.02
202	3.79	3.33	.02
512	3.83	3.54	.049
601	3.98	3.56	.03
Combined, mean (SD)	3.90 (0.09)	3.50 (0.10)	—^a^

^a^Not applicable.

## Discussion

### Principal Findings

In this study, we developed an innovative physician triage algorithm named the PPHR model. This model improves the accuracy of matching patients’ textual questions with physicians’ specialties and optimizes the ranking of candidates according to the physicians’ service performance. Consequently, the PPHR model may help increase both the efficiency and the quality of web-based medical services by recommending active physicians with the most appropriate specialties.

### Challenges and Solutions for Web-Based Triage Systems

Triage service is a preliminary service in medical diagnosis [29], serving as the first point of contact for patients in health care. It is crucial for improving the efficiency and precision of medical services. In offline outpatient clinics, patients’ choices are limited due to the physicians’ fixed schedules, especially if the appointment times cannot be changed. Therefore, triage is usually performed at the department level [30]. In contrast, web-based consultation services typically do not adhere to a fixed schedule, and all physicians can provide services on the web, so patients have a wider range of choices. However, the current triage systems have inherited the offline departmental recommendation form, which provides limited assistance to patients. In addition, due to the ongoing division of departments, the naming conventions of these departments have become confusing, resulting in possible overlap in disease areas that physicians specialize in across various departments. Furthermore, with the development of regional medical platforms [31], physicians from different regions and multiple hospitals may share the same web-based consultation platform, which complicates the supply of medical services. Therefore, it is imperative to develop a new type of physician recommendation system.

The construction of a triage system must first consider the matching of the physicians’ specialties with the patients’ medical needs, which is a prerequisite for the effective operation of web-based medical services [32]. The triage system needs to accurately create user profiles for physicians and patients, analyzing their characteristics, and achieve precise matching. Traditional triage systems typically use the profiles of professional-level physicians. These profiles mainly include the names of diseases and medical fields that physicians specialize in, which can be difficult to match with patients’ textual questions. Patients often ask their physicians questions using nonprofessional, colloquial descriptions of symptoms rather than precise disease names. Therefore, using professional descriptions to create physician profiles does not semantically match well with patients’ questions. Some studies have attempted to extract physicians’ features using textual questions from patients [5,11,12]. Our study draws on this approach, using natural language processing technology to build physician characteristics based on a large corpus of patient inquiries, thus constructing the profiles from the patient’s perspective and aligning more closely with patient needs.

In addition to expertise, the quality of service provided by physicians is equally important in ensuring effective web-based diagnosis and treatment. In web-based services, the quality of service is particularly reflected in the response time and rate, as well as the thoroughness of the content provided. Formally, response time and rate are obvious and accessible indicators. Previous research did not consider these indicators when developing triage systems. Therefore, we have included the consideration of the response rate in the scoring calculation of our model’s ranking. As there is a correlation between response rate and time, our results showed that this approach also significantly improved the recommended physicians’ response time.

### Feasibility and Potential Extensions of the Proposed Model

The most significant difference between web-based and offline medical consultations is the limited availability of data. When physicians cannot physically examine patients, the dialogue generated during the consultation becomes the primary source of usable information. Due to potential incompatibilities and lack of data sharing between web-based and offline systems [26], patients’ medical histories are often missing on most web-based consultation platforms, making it more challenging to extract patient characteristics. In terms of the quality of medical services, patients’ satisfaction with physicians is an indicator that can be referenced. However, the authenticity of satisfaction ratings on different platforms is not always reliable, as many users tend to habitually give positive feedback. Therefore, whether satisfaction ratings should be included in the model remains to be studied and verified. The PPHR model was designed to use minimal information to match physicians with patients. Despite the limited number of variables included, the advantage is that the algorithm is portable across different platforms, offering greater versatility and suitability for widespread adoption. Subsequently, different platforms can modify the model to suit their specific circumstances, including by incorporating past medical histories and satisfaction ratings. They can also adjust various hyperparameters within this framework, such as adjusting the weight of the response rates or setting a different number of recommendations to meet the needs of different platforms. In addition, the user interface can display information that the model used or disregarded, providing additional support for patient decision-making. Considering that some physicians may not be familiar with web-based platforms, it is also feasible to show indicators of their offline services. For instance, it is important to consider whether a physician has a sufficient number of in-person appointments and the level of satisfaction expressed by patients regarding those services. If a physician’s expertise is a good match for a specific type of consultation and they have outstanding offline reviews, despite not being highly active on the web, patients could consider switching to offline consultations during clinic hours.

### Real-World Application Challenges

Implementing the PPHR model in a real-world scenario presents a number of challenges. First and foremost, ethical issues related to privacy, consent, and confidentiality are major concerns. It is of the utmost importance that patients’ medical histories and personal information are handled with the utmost care, in accordance with the patients’ consent and in compliance with local and international laws. The PPHR model training process includes the pseudonymization or anonymization of data by removing or replacing personally identifiable information, as well as the use of SBERT for text vectorization, which transforms textual data into numerical vectors that represent the semantic meaning of the text but do not contain explicitly identifiable information, preventing the extraction of personal information directly from the vectors to protect individual privacy. It is also important to consider data confidentiality. Encrypting data at rest and in transit protects against unauthorized access. Using strong encryption standards, such as Advanced Encryption Standard for data at rest and Transport Layer Security for data in transit, can ensure that even if data are intercepted or accessed, they remain unreadable and secure. It is imperative to implement strong authentication mechanisms to verify the identity of users accessing the system; use multifactor authentication to add an extra layer of security; and implement role-based access control to ensure that users can access only the data relevant to their role, maintaining the principle of least privilege.

Integration with existing health care IT systems is another challenge. Many health care providers use legacy systems that may not be immediately compatible with newer models, such as PPHR. This requires the development of interfaces or middleware that can seamlessly connect the model to various health care IT infrastructures without disrupting existing workflows.

In addition, patient and physician acceptance is an integral part of the implementation process. Many users may be skeptical or resistant to changing traditional consultation methods. Educating both physicians and patients about the benefits of the PPHR model, such as increased efficiency; better physician-patient matching; and, ultimately, improved health care outcomes, is critical to facilitating adoption.

### Evolving Text Feature Extraction

Text embedding is a fundamental method for text feature extraction, where Doc2Vec is an effective means of implementing text embedding [33]. However, with the advent of transformer-based models, previous text embedding methods are gradually being replaced by SBERT in the industrial service sector. First, Doc2Vec provides a static embedding for each word, best used for tasks that can benefit from representations without the need for understanding word-context relationships [34]. SBERT provides dynamic contextual embeddings that allow for a deeper understanding of the meaning of words in context. It also has the ability to transfer knowledge and analyze subwords [35], which are essential for more complex language comprehension tasks.

Second, the computational efficiency of SBERT compared to traditional methods such as Doc2Vec is primarily influenced by its transformer architecture. Transformers take advantage of parallel processing, which significantly speeds up the training phase. However, they also tend to be resource-intensive, primarily due to the need for larger memory footprints to handle the contextual embeddings and underlying mechanisms. For the PPHR model, this means that there could be increased computational requirements, especially when processing a large corpus of patient queries or generating physician profiles.

Third, when considering the scalability of SBERT within the PPHR model for widespread use, several strategies can help mitigate potential challenges. Horizontal scaling, or adding more machines to spread the load, is a straightforward approach but can increase costs. More efficient strategies include the use of cloud-based services that offer dynamic scaling options to accommodate fluctuating demand without the need for constant, high-capacity infrastructure. Another key consideration is optimizing input sequences. By limiting the length of textual input without losing critical information, the PPHR model can reduce the processing required for each query, making the system more responsive. In addition, caching frequently accessed embeddings and using batch processing for embedding generation can significantly reduce the overall computational load.

It also indicates that as technology progresses, the underlying technical components of models must be regularly updated and refined to enhance the system’s overall efficiency. This is a real-world challenge that any web-based medical triage system in operation will encounter.

### Limitations and Future Directions

There are some limitations and further solutions. First, it is important to collect data from multiple sources. This study was limited to 1 hospital, which casts doubt on whether the findings are relevant in different contexts. To overcome this limitation, subsequent research should aim to collect information from various sources to evaluate the efficiency of the proposed algorithm. Second, there were some irrelevant contents in our data sets, for example, questions such as “Doctor, will you be available tomorrow? Where can I find you?” These business process–related questions are often mixed with medical questions describing symptoms and represent noises in the data set [36]. Even though preprocessing methods may reduce these noises, manual involvement might still be necessary to enhance the data quality. Third, a common limitation of deep neural networks [37] is the lack of a natural method to explain their predictive results, which makes it difficult to understand why specific samples are predicted to be similar. Models based on transformers make it very challenging to identify when unfair biases or spurious correlations might drive predictions. Therefore, we have introduced the involvement of physicians. If physicians could provide more information based on order details, such as scores based on the perspectives of professional suitability and willingness to accept orders, it might effectively improve the final performance of the model. Fourth, it is critical to regularly update the physicians’ professional information because this information changes over time. Relying on outdated data can result in less-than-ideal recommendations. To ensure that physicians’ profiles are up to date, a time range feature can be implemented. This feature automatically deletes data beyond the specified time range and periodically updates the model with only the latest data. This approach can improve the chances of making accurate recommendations for active physicians and reduce the chances of those still in training or changing areas of expertise. Fifth, obtaining valid feedback from patients and physicians is essential to validating the model’s benefits to patients in real-world settings on a larger scale. For instance, surveys can be conducted on patients’ use of the system, whether patients have adopted the system’s recommendations, and patients’ feedback on whether the system has been helpful. Observing changes in metrics such as the number of consultations for the same condition before and after using the system, comparing their outcomes, and examining the health economic effects of the system are also important. Finally, we were unfortunately only able to obtain textual data to develop the PPHR model. However, the triage system framework proposed in this paper has the potential to incorporate various types of data beyond text. It is possible to integrate multimodal information, such as text, images, audio, and video, using vector embedding techniques to create new vector features. On the basis of this, calculating similarities could potentially achieve more precise matching.

### Conclusions

This paper presents a PPHR model with response metrics that uses natural language processing techniques to tackle web-based medical triage tasks. The system filters out relevant physicians, aiding patients in finding the physicians who best suit their actual medical requirements. This approach has significant practical value and can be incorporated into various health website systems to enhance the quality of physician recommendations.

## Abbreviations

BERT: Bidirectional Encoder Representations from Transformers
PFB: patient feature–based
PPH: patient-physician hybrid
PPHR: patient-physician hybrid recommendation
SBERT: Sentence Bidirectional Encoder Representations from Transformers
TF-IDF: term frequency–inverse document frequency

## Data Availability

The data sets generated during and analyzed during this study are not publicly available due to privacy protection and data security regulations but are available from the corresponding author on reasonable request.
